# Editorial: Public hearing health: challenges and opportunities in the aftermath of the COVID-19 pandemic

**DOI:** 10.3389/fpubh.2023.1215248

**Published:** 2023-06-26

**Authors:** Panagiotis Katrakazas

**Affiliations:** Zelus_GR P.C., Athens, Greece

**Keywords:** public hearing health, low- and lower-middle-income countries, barriers and facilitative factors, COVID-19 aftermath, hearing loss prevention

Hearing health is a critical part of people's overall wellbeing (1). In addition to social isolation, sadness, and frustration, which often result in untreated hearing loss, there are many medical reasons for taking care of hearing (2). Hearing loss is associated with several serious health issues including diabetes and cardiovascular disease (3). It is also associated with a higher risk of falls (4). Many cases of tinnitus also occur in conjunction with hearing loss (5) and there is a higher risk of dementia in people with untreated hearing loss (6). A recent study showed that even people with mild hearing loss were twice as likely to develop dementia than those with normal hearing, and that the likelihood increases with higher degrees of hearing loss (7). The important and clear message is that hearing health is critical to the ability of hearing-affected people to remain socially connected and ensuring that their overall wellbeing is taken care of, even in unfortunate and unpredictable circumstances such as the recently experienced COVID-19 pandemic and especially in low- and middle-income countries.

This Research Topic titled “*Public hearing health: challenges and opportunities in the aftermath of the COVID-19 pandemic*” aims to enhance awareness and provide support to the hearing loss-affected community by identifying opportunities to increase access to and equity of better hearing healthcare, while addressing existing evidence gaps in hearing assessment, management, and prevention. The four selected articles in this Research Topic offer their respective authors' views, encompassing the factors, barriers, and challenges found mainly in low- and middle-income countries.

The first article from Grados-Espinoza et al. captures the mental health burden effect of the pandemic in terms of virtual education-related dissatisfaction among Peruvian medical students. Non-virtual and partial adaptation to such modalities, as well as stress levels, were found to be highly associated with students' satisfaction with virtual education, factors that should be considered as a future reference in the event of similar situations.

The second article refers to the development of a 24-item questionnaire derived by Nyarubeli et al. to assess the use of hearing protection devices among noise-exposed workers in manufacturing factories in Tanzania. The resulting questionnaire was preliminarily validated to reflect the real working environment, work culture, and traditions, and to accommodate the current technological situation in manufacturing factories in developing countries.

The final article, a systematic review by Oosthuizen et al. identifies limited research evidence in terms of combined hearing and vision screening programs, even after the pandemic event. There are still challenges to be addressed in terms of combined screening in hearing and vision, in an age-agnostic manner, where more feasibility and implementation research is required, especially in low- and middle-income countries.

The main interest in the selected articles is the focus on low- and middle-income countries, which are deemed to be of research interest. Gaps in terms of universal access and socio-economic and healthcare facility levels are a common factor identified across Peru (Grados-Espinoza et al.), Tanzania (Nyarubeli et al.), and beyond (Oosthuizen et al.). Hearing loss poses a considerable economic burden globally, estimated at $980 billion annually in terms of healthcare, education, loss of productivity, and societal costs (8). Robust and flexible economic evaluation methods are required to identify, enable, and optimize the potential of public hearing health facilitators to enable cost-effective prevention, diagnosis, and management of hearing loss. Optimizing hearing healthcare as part of a budget-constrained health system requires methods that can best inform joint research, reimbursement, and regulatory decision-making.

## Author contributions

PK: conceptualization, writing, editing, and review.
